# Upper Gastrointestinal Bleed Secondary to Duodenal Ischemia

**DOI:** 10.7759/cureus.7022

**Published:** 2020-02-17

**Authors:** Anupam K Gupta, Arye Lavin, Michael P Kucharik, Jordan Moseson

**Affiliations:** 1 Surgery, Charles E. Schmidt College of Medicine, Florida Atlantic University, Boca Raton, USA; 2 Internal Medicine, Charles E. Schmidt College of Medicine, Florida Atlantic University, Boca Raton, USA

**Keywords:** duodenal ischemia, management

## Abstract

We would like to present a rare case of postoperative duodenal ischemia managed conservatively. This is an 83-year-old female who underwent an elective costotransversectomy and discectomy. The surgery was complicated by hypotension. Postoperative recovery was complicated by episodes of diarrhea and melena with a hemoglobin drop. Esophagogastroduodenoscopy (EGD) performed revealed diffusely ischemic duodenal mucosa affecting both the first and second parts. Computed tomography angiography failed to find occlusion of blood supply. The patient was managed conservatively with fluids and hemodynamic support. The blood supply to the duodenum is highly collateral making ischemia here rare. In similar case reports of successfully conservatively managed duodenal ischemia, EGD was also performed due to similar rare presentations and diagnostic challenges/uncertainties, despite EGD currently not being considered a useful adjunct in the diagnosis of acute mesenteric ischemia. They were also managed conservatively with fluid replacement, bowel reset, and proper selection of current medications.

## Introduction

We would like to present a case of postoperative duodenal ischemia. Acute mesenteric ischemia (AMI) is characterized by interruption of the blood supply to varying portions of the small intestine leading to inflammation, ischemia, and eventually necrosis if untreated. The overall incidence is 0.09%-0.2% of all acute surgical admissions. Ischemia to the duodenum makes up a small fraction of this incidence rate as very few cases have been reported postoperatively [1]. This is an unusual finding which was managed in this case with fluid replacement, hydration, and hemodynamic support. Intestinal ischemia can affect anywhere in the small or large bowel. It is caused by any process that reduces intestinal blood flow including arterial occlusion, venous occlusion, vasoconstriction, and hypoperfusion. The likelihood of developing intestinal ischemia throughout the bowel depends on how adequate and sufficient the systemic perfusion and collateral circulation is in that area. Additionally, duration of ischemic insult and number of vessels affected could have an impact. The blood supply to the duodenum specifically is highly collateral making ischemia here rare, and therefore the management has not been well documented [2]. 

## Case presentation

This is an 83-year-old female former smoker with past medical history of atrial fibrillation, diabetes mellitus, hypertension, and a surgical history of lumbar/cervical spinal procedures four years prior. Her medications include metformin, sotalol, and aspirin (held prior to surgery). She presented with lower back pain and was found to have thoracic (12) and lumbar (1) disk herniation with spinal cord compression for which she underwent an elective costotransversectomy and discectomy from a posterior approach. Intraoperatively, the patient had blood loss of approximately 100 cc and suffered from hypotension, possibly due to anesthesia side effects or complications of her atrial fibrillation, with mean arterial blood pressure (MAP) as low as 40 mmHg (baseline MAP preoperative being 100 mmHg). The hypotension persisted in the immediate postoperative period (MAP, 40-60 mmHg) and was managed with fluid hydration and blood pressure medicine as needed. The Patient remained alert and oriented during her entire course in the hospital postoperatively.

On postoperative day 1, the patient had full neurological recovery but complained of several episodes of painless watery diarrhea. The lack of pain was likely due to postoperative analgesia. By postoperative day 2, she was having melanotic stools with a drop in her hemoglobin from a preoperative value of 11 gm% to 7.7 gm% which was associated with hypotension (MAP persisting 40-60 mmHg), as well as weakness and lethargy. The surgical site showed no evidence of bleeding, and her blood work did not reveal any alteration in coagulation. Her abdominal exam was soft throughout her hospital course. Her workup for stool studies was negative for an infective process including *Clostridium difficile* antigen.

**Figure 1:**
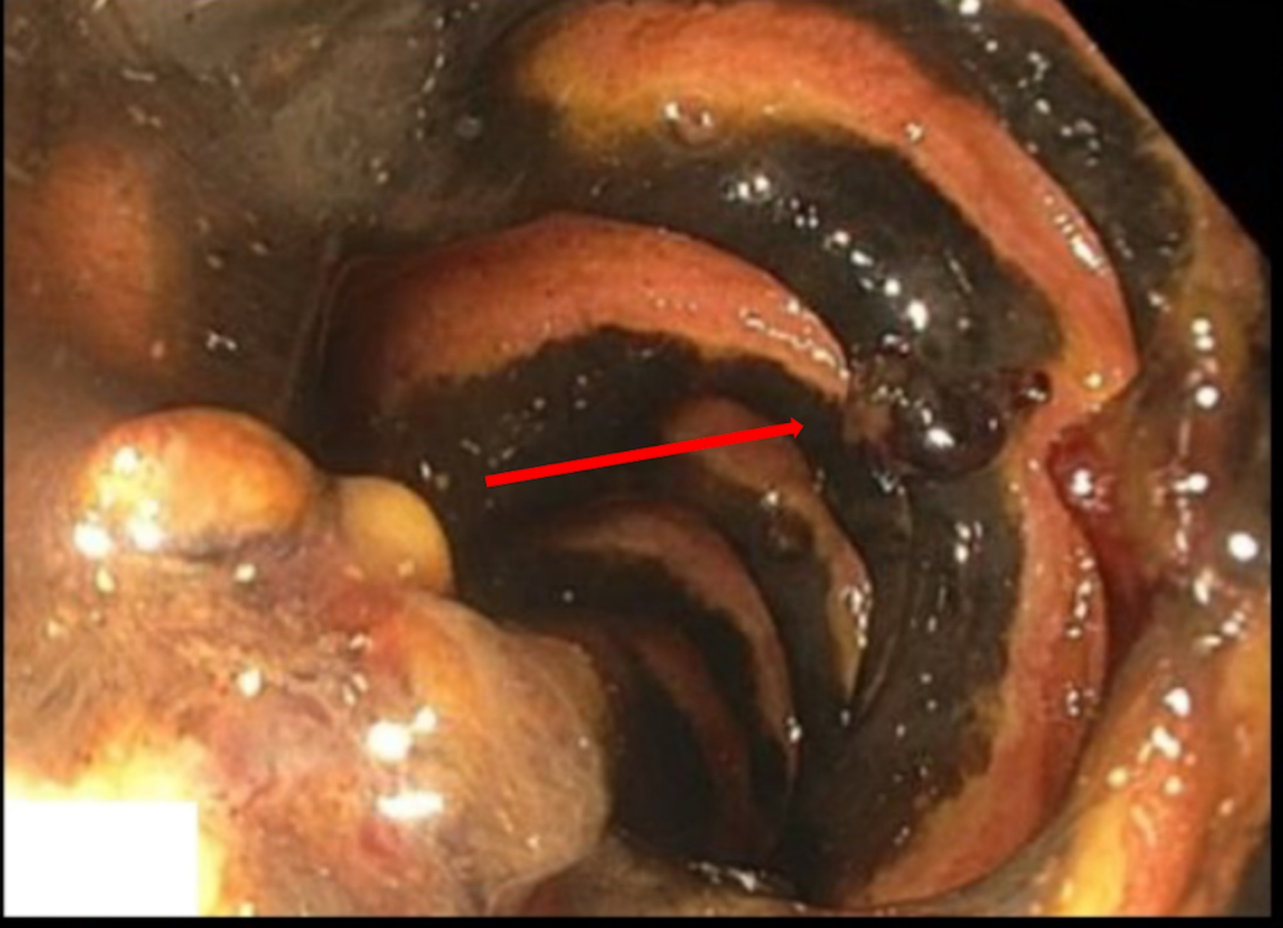
Esophagogastroduodenoscopy revealing patchy duodenal necrosis

**Figure 2:**
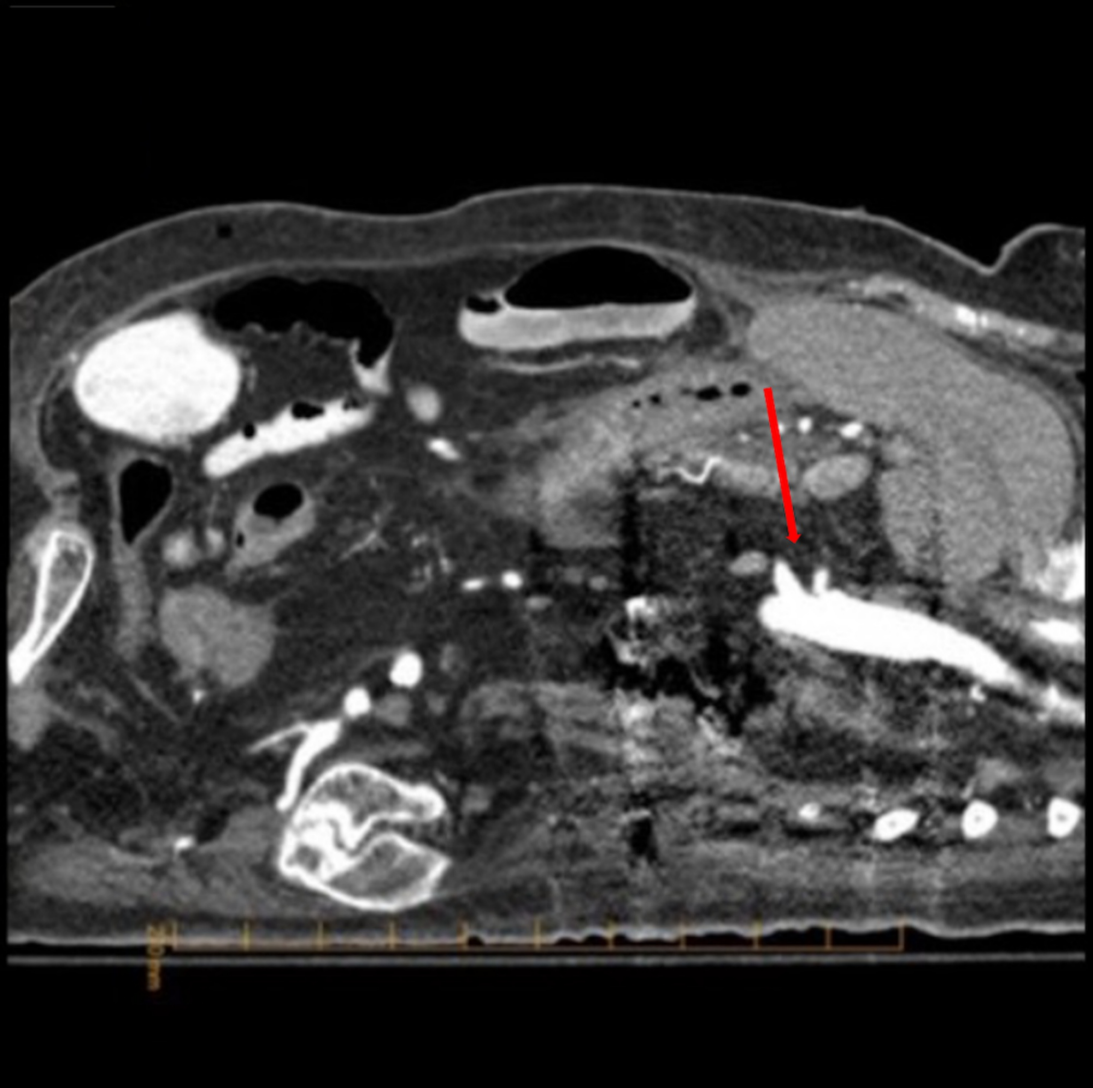
Computed tomography angiography showing a patent celiac and superior mesenteric artery

An esophageal duodenoscopy revealed diffusely ischemic duodenal mucosa affecting both the first and second parts (Figure 1). A computed tomography angiogram (CTA) of the abdomen did not reveal significant stenosis of the celiac artery (50%-60%) or superior mesenteric artery (50%-60%) (Figure 2).

**Figure 3:**
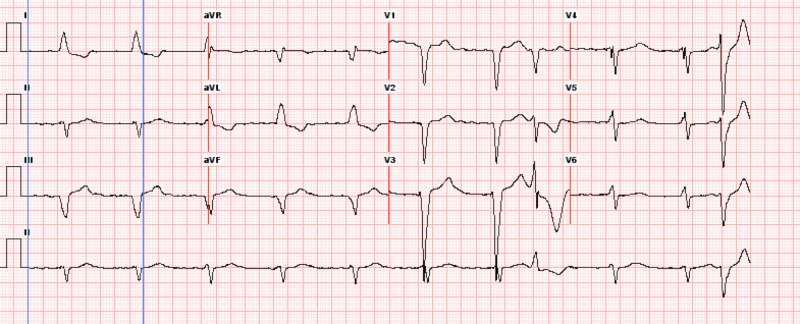
Electrocardiogram showing positive rate-controlled atrial fibrillation

The patient was hemodynamically supported to maintain MAP above 65 mmHg, with fluids along with avoidance of hypotensive and anticoagulant medication. Her cardiac workup during the process with electrocardiogram and echocardiogram was positive for rate-controlled atrial fibrillation (Figure 3). She converted back to normal sinus rhythm with diltiazem. On postoperative days 3-5, her hemoglobin stabilized between 7 and 8 gm% with a decrease in melena based on the nursing report of color of stool. Follow-up endoscopy revealed resolution of ischemia. She was discharged by post operative day 7 and has since been hemodynamically stable on her follow-up visits. 

## Discussion

The blood supply to the duodenum is highly collateral with the foregut vessels (celiac axis) and the midgut vessels (superior mesenteric artery) in the second portion with great variability [3]. In contrast, the “watershed zones” are usually the splenic flexure, the right colon, or rectosigmoid which can become ischemic [4].

While the most common cause of chronic mesenteric ischemia impacting the celiac axis or superior mesenteric artery is atherosclerotic disease, AMI is most commonly the result of superior mesenteric artery embolization, superior mesenteric artery thrombosis, non-occlusive mesenteric ischemia, or acute mesenteric venous thrombosis which does not typically affect the duodenum [5]. Duodenal ischemia has been reported in the literature secondary to occlusive disease, abdominal aortic aneurysms, complications of percutaneous endoscopic gastrostomy tube placement, endoscopic retrograde cholangiopancreatography, and transarterial chemoembolization [6]. However in this case, the patient’s operative/postoperative course was complicated by hypotension leading to duodenal mucosal ischemia from hypoperfusion.

This patient demonstrated duodenal ischemia confirmed on esophageal duodenoscopy with CTA negative for significant stenosis of the celiomesenteric trunk. In prior reports, duodenal ischemia was managed by providing adequate hemodynamic support, without any need for surgical intervention. Had clinical, radiographic, or laboratory parameters indicated intestinal infarction or perforation, abdominal exploration would have been urgently required to remove necrotic or gangrenous portions of bowel and prevent progression to sepsis and preserve bowel function. The ischemia in these reports was discovered using esophageal duodenoscopy [7,8]. Our patient was similarly managed without surgical intervention with fluid replacement, bowel rest, and proper hemodynamic support. 

## Conclusions

It is unusual to have duodenal ischemia due to the extensive blood supply and collaterals. It can be diagnosed with esophageal duodenoscopy to determine the extent of the ischemia. It is important to consider vascular, cardioembolic, infections, and medication as causes of the ischemia. Ischemic duodenum secondary to a low flow state is an unusual entity and diagnosed by exclusion of other causes of ischemia and including an esophageal duodenoscopy in the workup. Initial management should be symptomatic and treated with hemodynamic and medical support.
